# Comparison of Pulmonary Artery Pressure Measurement With Doppler Echocardiography or With Right Heart Catheterization in Patients With Congenital Heart Disease

**DOI:** 10.3389/fped.2019.00421

**Published:** 2019-10-18

**Authors:** Dan Yin, Ying Wang, Min Zheng, Huijing Wei, Mi Li, Tiewei Lv, Yonghong Bai, Jie Tian, Xiaoyun Wu

**Affiliations:** ^1^Ministry of Education Key Laboratory of Child Development and Disorders, Chongqing, China; ^2^China International Science and Technology Cooperation Base of Child Development and Critical Disorders, Chongqing, China; ^3^Department of Cardiovascular Medicine, Children's Hospital of Chongqing Medical University, Chongqing, China; ^4^National Clinical Research Center for Child Health and Disorders, Chongqing, China; ^5^Chongqing Key Laboratory of Pediatrics, Chongqing, China

**Keywords:** pediatrics, congenital heart disease, echocardiography, systolic pulmonary artery pressure, right heart catheterization

## Abstract

**Background:** Doppler echocardiography (D-ECHO) is a commonly used imaging tool for both diagnosis and follow-up examination of congenital heart disease (CHD). The goal of this study is to evaluate the accuracy of D-ECHO as used to measure an estimate sPAP in pediatric patients with CHD.

**Methods:** A prospective study in 397 pediatric patients with CHD has been carried out to compare estimate sPAP measured with D-ECHO to that measured with right heart catheterization (RHC). Pearson correlation analyses were used to calculate the correlation coefficients between RHC and D-ECHO. Bland-Altman analyses were carried out to assess the agreement between the two methods.

**Results:** Our data have demonstrated a significant underestimation of sPAP by D-ECHO compared to that by RHC. A strong correlation (*r* = 0.957, *p* < 0.01) was found between sPAP (36.1 ± 14.9 mmHg) and RVSP (36.0 ± 14.5 mmHg) measured with RHC. However, a relatively weak correlation (*r* = 0.219, *p* < 0.01) was observed between sPAP (36.1 ± 14.9 mmHg) measured during RHC and sPAP (28.7 ± 9.7 mmHg) as estimated using D-ECHO. The Bland-Altman analysis demonstrated that the bias for D-ECHO sPAP estimates was 6.6 mmHg with 95% limits of agreement ranging from −23.6 to 36.8 mmHg. A total of 57.5% of D-ECHO measurements were found to be accurate, with accuracy predefined as 95% of agreement within ±10 mmHg for sPAP estimates.

**Conclusions:** sPAP measured with D-ECHO may be underestimated in pediatric patients with CHD.

## Introduction

Pulmonary artery hypertension (PAH) is defined as a mean pulmonary artery pressure (mPAP) ≥25 mmHg at rest with a right heart catheterization (RHC). Some primary PAH cases are associated with congenital heart disease (PAH-CHD) with a systemic-to-pulmonary shunt (1). The prevalence of PAH-CHD cases is about 1.6 ~ 12.5/1,000,000 in general population. However, ~6.5–10% of adult CHD patients are estimated to develop PAH (2). A study carried out in 2007 has demonstrated that PAH-CHD patients develop into Eisenmenger' s syndrome (ES) and had an average survival rate of 32.5 ± 16 years, and the survival rates in patients of 30, 40, and 50 years old are 75, 70, and 50%, respectively (3). An accurate assessment of pulmonary arterial pressure (PAP) is important in order to grasp the operative indications of CHD and improve the patient prognosis. Currently, RHC is a gold standard for PAP assessment and has been demonstrated to be a critical tool for the evaluation of surgical outcomes in CHD cases. However, since RHC is an invasive examination and is expensive and difficult to perform, a non-invasive, accurate PAP assessment is therefore critical for CHD diagnosis and treatment. Doppler echocardiography (D-ECHO) is currently the most commonly used non-invasive imaging tool for CHD diagnoses and follow-up studies. Furthermore, D-ECHO is also used as a non-invasive screening tool for the assessment of PAP. However, the accuracy of the estimated systolic pulmonary artery pressure (sPAP) measured by D-ECHO is in question in various clinical studies. Overall, a correlation between sPAP estimated by D-ECHO and sPAP measured by RHC was reported that ranged from *r* = 0.31 to *r* = 0.99 (4). Fisher et al. (5) studied in 65 adults Pulmonary hypertension (PH) patients and D'Alto et al. (6) studied in 161 adults patients who were suspected PH and Rich et al. (7) carried out their study in 23 PAH patients demonstrated that estimated sPAP by D-ECHO was inaccurate. However, the pediatric data regarding the accuracy of D-ECHO measurement compared to RHC examination of sPAP in pediatric patients with CHD is very limited. The goal of this study is to evaluate the accuracy of the estimated sPAP measured by D-ECHO compared with that measured by RHC in pediatric patients with CHD.

## Patients and Methods

### Patients

The research project was authorized and approved by the Ethics Committee of Children's Hospital of Chongqing Medical University. Written informed consent was obtained from all study subjects in accordance with the Declaration of Helsinki. In the case of patients under 16 years old, written informed consent was provided by their guardians. We prospectively enrolled 397 CHD children from the Pediatric Cardiology Department of Children's Hospital of Chongqing Medical University from April 2016 to April 2017. Inclusion criteria: (1) Children diagnosed with simple ASD/VSD/PAD from the Pediatric Cardiology Department of Children's Hospital of Chongqing Medical University from April 2016 to April 2017. (2) Enrolled subjects were confirmed without right ventricular outflow tract (RVOT) obstruction. (3) Enrolled subjects have no family history of pulmonary hypertension. (4) The formal evaluation of RHC were carried out within 48 h following the D-ECHO. (5) Written informed consent was obtained from all study subjects. Exclusion criteria: (1) Children diagnosed with non-simple ASD/VSD/PAD. (2) Enrolled subjects were observed with right ventricular outflow tract (RVOT) obstruction. (3) Enrolled subjects have family history of pulmonary hypertension. (4) The formal evaluation of RHC were carried out over 48 h following the D-ECHO. (5) Subjects did not sign written informed consent. Of the CHD children enrolled, 167 patients had only patent ductus arteriosus (PDA), 122 patients had only atrial septal defects (ASD), and 108 patients had only ventricular septal defects (VSD). A total of 163 of the enrolled patients were males and 234 were females, with an average age of 39.8 months and an age range from 5 to 187 months, with 90.2% being under 72 months. No enrolled patients reported a family history of PAH. The formal evaluation of D-ECHO and RHC were carried out within 48 h of each other, and RHC was typically performed 12–36 h following the D-ECHO. Operators worked independently for each study and were blinded to the study results.

### Doppler Echocardiography

The D-ECHO and complete two-dimensional were recorded using ultrasound systems (Philips, ie33). Subjects younger than 3 years of age were sedated by a professional anesthesiologist prior to the examination. Supine position was placed before testing, and normative four-chamber and parasternal views were obtained by two independent operators. Any discrepancies were resolved through discussion with a third operator in order to reach a final consensus. The right atrial pressure (RAP) was added to the trans-tricuspid pressure gradient (TPG) in order to estimate the right ventricular systolic pressure (RVSP) (8–10) (RVSP = RAP + TPG), and no one was observed with right ventricular outflow obstruction. The modified Bernoulli equation was used to calculate TPG (TPG (mmHg) = 4Vmax^2^), where V represents the peak systolic velocity (m/sec) of tricuspid regurgitation (TR) in the parasternal short axis and the four-chamber apical views. According to the size of the right atrium and the degree of tricuspid regurgitation, RAP was estimated as 5 or 10 mmHg (11). If the size of the right atrium remains constant and tricuspid regurgitation is mild, the RAP is estimated to be 5 mmHg, otherwise it is estimated to be 10 mmHg. Without right ventricular outflow tract (RVOT) obstruction, RVSP is considered to be equal to sPAP. Indicators of measuring cardiac function and morphology in this study include: right atrial diameter (RA), right ventricular internal diameter (RV), ejection fraction (EF), main pulmonary artery internal diameter (MPA), and tricuspid annular plane systolic excursion (TAPSE).

### Right Heart Catheterization

With the patient under general anesthesia, a multi-lumen thermo-dilution catheter was inserted via the right femoral vein (RFV) and positioned 5–10 cm distal to the pulmonary valve. We continuously record the waveform of pulmonary artery pressure, select three relatively stable periods, and take the average.

### Statistical Analysis

SPSS version 22.0 was used to carry out statistical analyses in this study. Mean ± SD was calculated to present continuous data, while the percent and frequency were used to present categorical data. Chi-square test and Student's *t*-test were used to analyze data when appropriate. Statistical significance was defined as a two-side value of *p* < 0.05. Pearson correlation analyses were used to calculate the correlation coefficients between RHC and D-ECHO. However, a good correlation does not indicate a good agreement, nor does it indicate that one test can reliably replace another. Therefore, we used the Bland-Altman analyses to accurately assess the agreement between the two methods (12). 95% limits of agreement within ± 10 mmHg for sPAP estimates were defined as accurate in this study (7).

## Results

**Figure 1:**
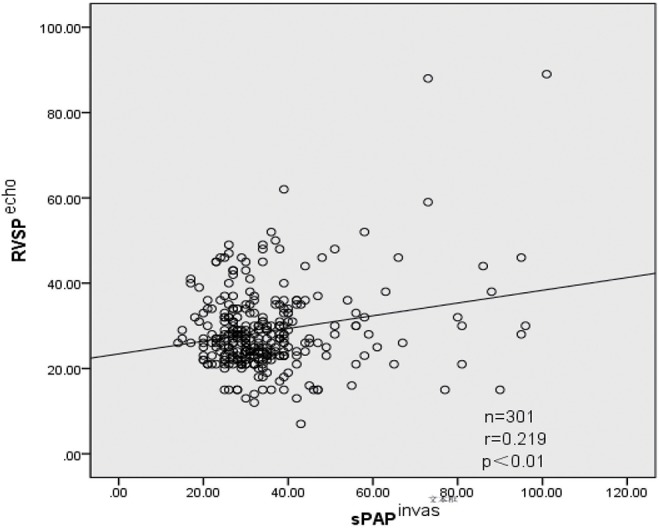
Relationship between RVSP estimated by D-ECHO and sPAP observed by RHC (*r* = 0.219, *p* < 0.01). Echo, measured by Doppler echocardiographic; invas, invasive measurement by right-heart catheterization; r, correlation coefficient (Pearson); sPAP, systolic pulmonary artery pressure; RVSP, right ventricular systolic pressure.

**Figure 2:**
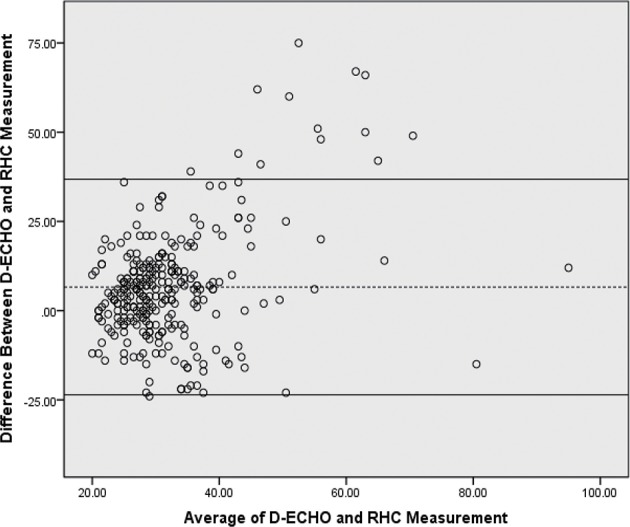
Bland-Altman plot of sPAP estimated by Doppler echocardiographic and RHC. The bias was 6.6 mmHg and the 95% limits of agreement were 23.6–36.8 mmHg. Dotted line, bias; solidline curve, upper and lower limits of agreement. D-ECHO, Doppler echocardiography; sPAP, pulmonary artery systolic pressure; RHC, right-heart catheterization.

**Figure 3:**
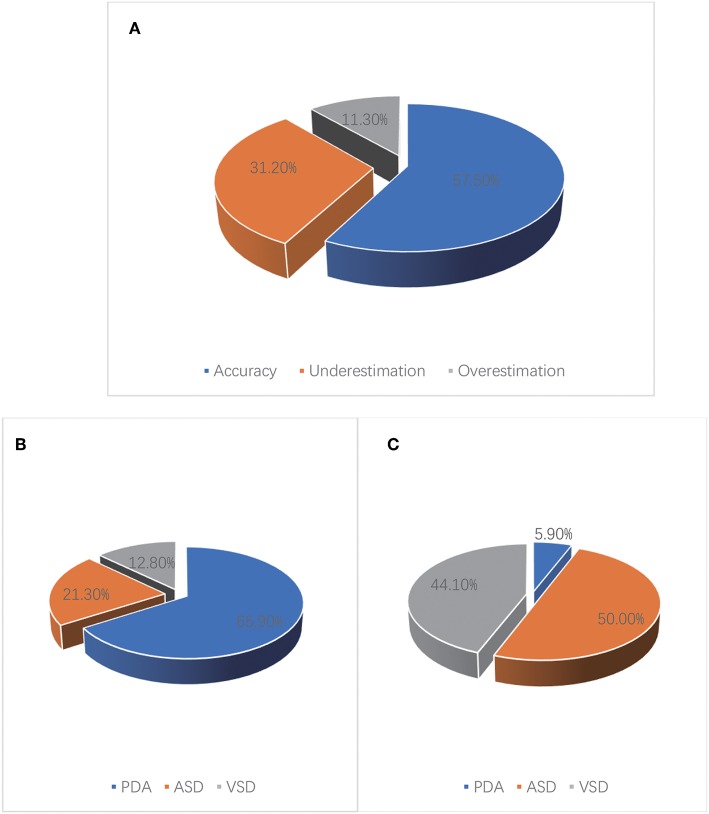
Accuracy of sPAP estimated by D-ECHO in 301 children with CHD. **(A)** A total of 57.5% of D-ECHO estimates were found to be accurate, 31.2% of D-ECHO estimates were found to be underestimated, 11.3% of D-ECHO estimates were found to be overestimated. **(B)** In patients with underestimated pressure, it was found that PDA accounted for 65.9%, ASD accounted for 21.3%, and VSD accounted for 12.8%. **(C)** In patients with overestimated pressure, it was found that PDA accounted for 5.9%, ASD accounted for 50.0%, and VSD accounted for 44.1%.

A total of 397 CHD pediatric patients were prospectively enrolled in this study. Of the children enrolled, 301 estimated sPAP using both D-ECHO and RHC. Patient baseline characteristics and summary statistics for hemodynamic measurements obtained from RHC and D-ECHO are presented in Table 1. A wide range of RVSP (10–97 mmHg), and sPAP (14–101 mmHg) was measured during RHC. And mean RAP (mRAP) during RHC was 7.4 mmHg. A strong correlation observed between sPAP (36.1 ± 14.9 mmHg) and RVSP (36.0 ± 14.5 mmHg) during RHC (*r* = 0.957, *p* < 0.01). However, A relatively weak correlation (*r* = 0.219, *p* < 0.01) was observed between sPAP (36.1 ± 14.9 mmHg) during RHC and sPAP (28.7 ± 9.7 mmHg), which was estimated by D-ECHO (Figure 1). Using the Bland-Altman analysis, a bias for sPAP D-ECHO estimates was determined to be 6.6 mmHg with 95% limits of agreement ranging from −23.6 to 36.8 mmHg (Figure 2). A total of 57.5% of D-ECHO estimates were found to be accurate, with accuracy predefined as 95% of agreement within ±10 mmHg for sPAP estimates. In patients with underestimated pressure, it was found that PDA accounted for 65.9%, ASD accounted for 21.3%, and VSD accounted for 12.8%. However, it was found that PDA accounted for 5.9%, ASD accounted for 50.0%, and VSD accounted for 44.1% in patients with overestimated pressure (Figure 3). A total of 42 out of 94 patients (44.7%) had pressure underestimates that were >20 mmHg, while 10 out of 34 patients (29.4%) had pressure overestimates that were >20 mmHg. In addition, we also observed that the underestimation was greater than the overestimation (−23.1 ± 14.4 vs. 16.2 ± 4.1 mmHg; *P* < 0.01).

**Table 1:** The baseline characteristics and summary statistics for hemodynamic obtained during RHC and D-ECHO of the patients.

Male, *n* (%)	163 (41.1%)
Age (Mean ± SD, month)	39.8 ± 28.6
PDA, *n* (%)	167 (42.1%)
ASD, *n* (%)	122 (30.7%)
VSD, *n* (%)	108 (27.2%)
**Doppler echocardiography measurements**	
RVSP_D−ECHO_ = RAP+TPG (mmHg)	28.7 ± 9.7
RVSP_D−ECHO_ ≤ 30 mmHg	208 (69.1%)
RVSP_D−ECHO_ 31–45 mmHg	74 (24.6%)
RVSP_D−ECHO_ 46–70 mmHg	17 (5.6%)
RVSP_D−ECHO_ >71 mmHg	2 (0.7%)
RAP_D−ECHO_ (mmHg)	5.5 ± 1.5 mmHg
Right ventricular length diameter (mm)	48.6 ± 8.2
Right ventricular horizontal diameter (mm)	28.7 ± 7.0
Right ventricular anteroposterior (mm)	13.2 ± 3.0
Right atrial length diamete (mm)	18.3 ± 4.3
Right atrial horizontal diameter (mm)	20.6 ± 4.7
MPA (mm)	18.3 ± 3.2
TAPSE (mm)	18.7 ± 3.5
EF (%)	63.2 ± 9.4
**Right-heart catheterization hemodynamics**	
sRAP_RHC_ (mmHg)	13.6 ± 4.0
d RAP_RHC_ (mmHg)	4.3 ±3.9
m RAP_RHC_(mmHg)	7.4 ±3.3
RVSP_RHC_ (mmHg)	36.0 ± 14.5
sPAP (mmHg)	36.1 ± 14.9
sPAP <30 mmHg	136 (45.2)
sPAP 31–45 mmHg	126 (41.9)
sPAP 46–70 mmHg	26 (8.6)
sPAP>70 mmHg	13 (4.3)
dPAP (mmHg)	16.2 ± 9.9
mPAP (mmHg)	22.8 ± 11.1

*PDA, patent ductus arteriosus; ASD, atrial septal defects; VSD, ventricular septal defects; D-ECHO, Doppler echocardiography; RVSP_D−ECHO_, right ventricular systolic pressure estimated by Doppler echocardiography; RAP, right atrial pressure; TPG, transtricuspid pressure gradient; RAP_D−ECHO_, right atrial pressure estimated by Doppler echocardiography; MPA, main pulmonary artery; TAPSE, tricuspid annular plane systolic excursion; EF, ejection fraction; sRAP_RHC_, systolic right atrial pressure measured by right-heart catheterization; dRAP_RHC_, right atrial diastolic pressure measured by right-heart catheterization; mRAP_RHC_, mean right atrial pressure measured by right-heart catheterization; RVSP_RHC_, right atrial pressure measured by right-heart catheterization; sPAP, systolic pulmonary artery pressure; dPAP, pulmonary artery diastolic pressure; mPAP, mean pulmonary arterial pressure*.

**Figure 4:**
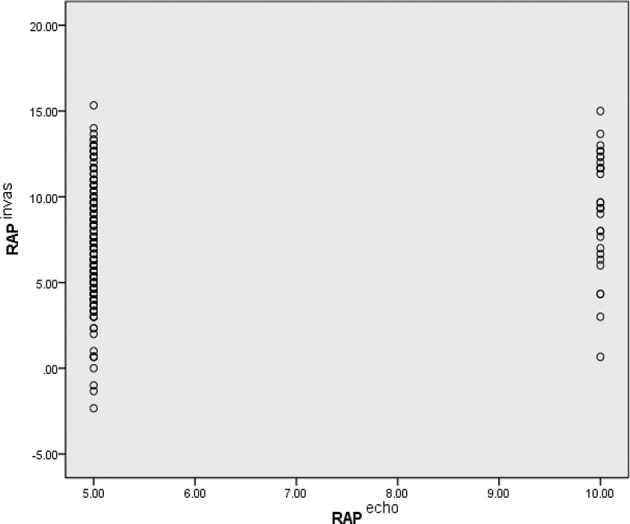
Comparison of RAP as estimated by D-ECHO and RHC. Echo, measured by Doppler echocardiographic; invas, invasive measurement by RHC; RAP, right atrial pressure.

Figure 4 depicts the RAP distribution estimated using D-ECHO and measured using RHC. RAP was estimated using D-ECHO and RHC in a total of 244 subjects. In the mass, RAP estimated using D-ECHO was found to be lower than when measured using RHC (5.5 ± 1.5 vs. 7.4 ± 3.3 mmHg; *p* < 0.01). The bias of D-ECHO estimated was 8.2 mmHg, with 95% limits of agreement ranging from 0.2 to 16.2 mmHg.

For clinical perspective, PAH is classified as severe if sPAP is >70 mmHg, moderate if sPAP is between 46 and 70 mmHg, mild if sPAP is between 31 and 45 mmHg, and normal if sPAP is between 15 and 30 mmHg. Upon comparing sPAP measurements made using RHC vs. those estimated using D-ECHO, we observed that only 43.5% of subjects were in the same diagnostic category. A total of 40.9% of the subjects had sPAP measurements underestimated by D-ECHO. Of these underestimates, 32.2% were in one diagnostic category and 8.6% were in more than one diagnostic category. In contrast, only 15.7% of subjects had sPAP overestimates made by D-ECHO and 14.0% of these overestimates were in one diagnostic category (Table 2). We calculated the false positive and false negative of PAH if PA pressures are based on D-ECHO (Table 3).

**Table 2:** Comparison of PAH diagnostic category according to sPAP measured by RHC vs. sPAP estimated by D-ECHO.

	**Diagnostic category error**	**%**
Underestimate	3	2.0%
	2	6.6%
	1	32.2%
Same	0	43.5%
Overestimate	1	14.0%
	2	1.7%

*Classify PAH as severe if the sPAP was more than 70 mmHg, moderate (46–70 mmHg), mild (31–45 mmHg), and normal (15–30 mmHg). 0, same diagnostic category; 1, 1 category of misclassification; 2, 2 category of misclassification; 3, 3 category of misclassification. %, Percentage of this category in total number of persons*.

**Table 3:** The false positive and false negative of PAH if PA pressures are based on D-ECHO.

	**Patients**	**Normal**	**Total**
Positive	53	40	93
Negative	113	95	208
Total	166	135	301

*PAH, sPAP > 30 mmHg estimated by RHC or RVSP*.

True positive, 31.9%.

True negative, 70.4%.

False positive, 29.6%.

*False negative, 68.1%*.

## Discussion

In this study, we have evaluated the accuracy of estimated sPAP measured by D-ECHO compared to that measured by RHC in pediatric patients with CHD at a single medical center. Both tests were carried out sequentially within 36 h. The subject tested included only one single CHD, i.e., ASD, VSD, or PDA. We observed a significant difference between sPAP estimated by D-ECHO and that measured by RHC. With assessments made by Band-Altman analyses, D-ECHO tended to cause either an underestimate or an overestimate sPAP, with most underestimated cases.

In 1984, due to a good correlation between sPAP estimated by D-ECHO and sPAP measured by RHC (*r* = 0.93, *p* < 0.01), Yock and Pope (13) first reported that the tricuspid gradient method could provide us with an accurate and widely applicable tool for non-invasively measurement of sPAP. Numerous subsequent studies (14–16) support this conclusion, and the sPAP estimates non-invasively measured by D-ECHO became widely accepted (1, 17). Non-invasive diagnostic methods are understandably attractive to both doctors and patients. However, the good correlation observed between D-ECHO and RHC does not necessarily indicate a good agreement and does not mean that one test can reliably replace the other.

Our study demonstrated a relatively weak correlation between D-ECHO sPAP estimates and RHC sPAP measurements in children with CHD. Overall, a correlation was reported that ranged from *r* = 0.31 to *r* = 0.99 (4). Using Bland-Altman analyses to assess the level of agreement between RHC and D-ECHO, we demonstrated that 41.3% of D-ECHO measurements were not accurate. Both overestimates and underestimates were found in sPAP estimates using D-ECHO, with underestimates found to be more common. This is in consistent with what reported by other studies (5, 7). Due to the variability of pulmonary vascular resistance (PVR) and cardiac output (CO), sPAP can fluctuate widely within a single day (18, 19). In our study, both invasive and non-invasive measurements were carried out within 36 h. However, Fisher et al. (5) and D'Alto et al. (6) limited the interval between invasive and non-invasive measurements to 1 h, while Rich et al. (7) carried out their study in 23 PAH patients and performed simultaneous RHC and D-ECHO measurements of sPAP and demonstrated that estimated sPAP by D-ECHO was inaccurate. It was also found that carrying out sPAP D-ECHO estimates and RHC measurements simultaneously did not significantly improve the agreement (20). Interestingly, it has been demonstrated that PDA accounts for most of underestimations, while overestimation dominates ASD and VSD. Zhang et al. (20) carried out a study in 257 patients only with ASD and demonstrated that D-ECHO resulted in overestimates of sPAP. An accurate assessment of sPAP is critical for proper treatment and prognosis. Whether sPAP is overestimated or underestimated, these measurements are not conducive for the diagnosis and management of PAH patients. According to the modified Bernoulli equation [TPG (mmHg) = 4Vmax^2^], the first condition to accurately estimate sPAP depends on the presence of tricuspid regurgitation and the ability to achieve the correct peak velocity of the tricuspid regurgitation jet. Therefore, in the absence of TR, D-ECHO cannot accurately estimate sPAP, or underestimates sPAP when TR is insufficient. In addition, estimated intra-cardiac pressure at the end of the exhalation period often leads to obvious pressure underestimations, which is particularly reflected in patients with obesity and lung disease (21). Third, if the TR jet and the Doppler beam are not perfectly aligned in a parallel arrangement, there is an intercept angle between them, resulting in inaccurate TPG measurements (and underestimation is common) (22). While the factors described above are avoided, the modified Bernoulli equation still retains limitations. The modified Bernoulli equation ignores the effects of the fluid viscosity and inertia. In addition, it assumes that the potential energy (right ventricular pressure) is converted completely into kinetic energy (TR), which could lead to an underestimation. Because the velocity in the equation is squared, the error associated with the measurement of speed has a greater influence on the result when the reflux velocity increases (the right ventricular pressure increases). The TR insufficiency could be the reason that explains why PDA patients account for 65.9% of underestimations. Right ventricle overload and pulmonary over circulation in ASD and VSD patients could be another reason that ASD and VSD patients account for >90% of overestimations. Left-to-right shunt in VSD patients may also be mixed into TR, resulting in a high peak systolic velocity of TR and elevated TPG.

Inaccurate RAP estimates could also result in inaccurate sPAP estimates. According to the size of the right atrium and the degree of tricuspid regurgitation, RAP estimated as 5 or 10 mmHg (11). In adults, ASE recommendations inferior vena cava (IVC) size along with its respirophasic variation to estimate RAP in most common: IVC diameter <2.1 cm that collapses >50% with a sniff suggests normal RAP of 3 mmHg (range, 0–5 mmHg), whereas an IVC diameter >2.1 cm that collapses <50% with a sniff suggests a high RAP in the 15 mmHg (range, 10–20 mmHg). In scenarios that the IVC diameter and collapse do not fit this paradigm, an intermediate value of 8 mmHg (range, 5–10 mmHg) may be used (23). However, there currently exists no standard for children. Brennan et al. (24) systematically evaluated IVC echocardiographic imaging to estimate RAP in 102 patients undergoing RHC. That study demonstrated that it is inexactitude. In a similar case, Fisher et al. (5) and Rich et al. (7) demonstrated a same conclusion when they showed a wide spread between values obtained using D-ECHO and RHC estimated using RAP. In 2015, Arya et al. (11) and colleagues demonstrate the utility of certain echocardiographic parameters for the assessment of RAP in a pediatric and young adult population. This study demonstrated that among various echocardiographic parameters, RAV had a modest correlation with mean RAP in pediatric and young adult patients. The long-axis IVC max and tricuspid E wave also demonstrated weak relationships with mean RAP in this population. This study may be helpful in estimating RAP by non-invasive method. For all that, the best method for the accurate estimation of RAP needs to be studied further.

### Limitation

Some limitations exist for this study due to the local medical and economic conditions. For example, some important measurements could not be performed, such as Qp/Qs, PVR, and PCWP. We understand the importance of these parameters in the diagnosis. The ratio between pulmonary (Qp) and systemic (Qs) flow indicates the existence of some type of short circuit between two circulations, either intra-, and extra-cardiac circulations. After the birth, under normal conditions the pulmonary and systemic expenditure are practically the same, and the ratio Qp/Qs = 1. If the quotient of Qp/Qs is >1, which means the short circuit will be from the circulation systemic to pulmonary, and if <1, from pulmonary toward the systemic circulation. In simple intracardiac shunts, the interpretation of the data is immediate. The high Qp/Qs means an important short circuit, and the Qp/Qs <1 suggests an Eisenmenger syndrome. In all other situations (persistent duct, pulmonary atresia, extracardiac shunts, univentricular correction, etc.), the measurements must be obtained and the data interpreted according to the pathophysiology. Qp and Qs are very important for judging the direction of shunt and pulmonary circulation. In addition, PVR represents pulmonary vascular resistance and PCWP represents pulmonary capillary wedge pressure, these two values are important indicators for diagnosing PAH. The diagnostic criteria are mean PA pressure >25 mmHg at rest and pulmonary capillary wedge pressure <15 mmHg and elevated pulmonary vascular resistance >3 WU or >3 WUm2 for pediatric PAH. We regret that we did not have these measurements. Although with the limitations, this study has some values in the field since the purpose of this study was to evaluate the accuracy of pulmonary artery systolic pressure estimated by ultrasound and catheter measurements. Ultrasound estimation is simple and easy, and cardiac catheterization was performed when ultrasound estimation found significant increase in pulmonary artery systolic pressure. This approach may be practical for some areas with less developed economies and with insufficient medical facilities.

## Conclusions

While we concluded from this study that sPAP/RAP values obtained using D-ECHO are underestimated, D-ECHO remains an important technique for the evaluation and screening of PAH. However, clinical workers should not overly reliant alone on D-ECHO pressure estimates for patients with suspected PAH. Cautious should be taken on evaluating D-ECHO data that support pulmonary hypertension, including RV enlargement, hypertrophy of the right ventricular wall, widening of the main pulmonary artery, changes in ventricular septal morphology, acceleration of pulmonary regurgitation velocity, and the shortening of acceleration time of the right ventricular outflow tract forward flow. In addition, multiple index parameters must be applied in combination with each other and each should be analyzed comprehensively in order to improve the accuracy of the measurements.

## Data Availability Statement

The datasets generated for this study are available on request to the corresponding author.

## Disclosure

This manuscript has been thoroughly edited by a native English speaker from an editing company. Editing Certificate will be provided upon request.

## Author Contributions

XW, YW, and DY: experimental design, data collection, and article writing. JT, TL, ML, HW, MZ, and YB: experimental design and data collection.

### Conflict of Interest

The authors declare that the research was conducted in the absence of any commercial or financial relationships that could be construed as a potential conflict of interest.
